# Critical analysis of molecular tests in indeterminate thyroid nodules

**DOI:** 10.20945/2359-3997000000095

**Published:** 2018-10-01

**Authors:** Debora L. S. Danilovic, Suemi Marui

**Affiliations:** 1 Universidade de São Paulo Universidade de São Paulo Faculdade de Medicina Hospital das Clínicas São Paulo SP Brasil Laboratório de Endocrinologia Celular e Molecular (LIM25), Hospital das Clínicas, Faculdade de Medicina, Universidade de São Paulo (HCFMUSP), São Paulo, SP, Brasil; 2 Universidade de São Paulo Universidade de São Paulo Faculdade de Medicina Instituto do Câncer do Estado de São Paulo São Paulo SP Brasil Instituto do Câncer do Estado de São Paulo, Faculdade de Medicina, Universidade de São Paulo (FMUSP), São Paulo, SP, Brasil

The prevalence of thyroid nodules has increased in the last decades, mostly due to the widespread use of cervical imaging for investigation of pathologies not related to thyroid (incidentalomas) and, eventually, thyroid imaging screening for individuals at no risk for thyroid disease. Nevertheless, thyroid carcinomas correspond to 5%-15% of thyroid nodules and the failure in identifying benign nodules in asymptomatic patients usually leads to unnecessary thyroidectomies. Ultrasonography and fine needle aspiration biopsy (FNAB) contribute to preoperative diagnosis, but indeterminate cytology still represents 20% to 30% of diagnosis, namely Bethesda III, IV and V, with rates of malignancy reported as 10%-30%, 25%-40% and 50%-75%, respectively (1). In this context, the recent knowledge of molecular abnormalities related to thyroid cancer has been used to improve patient outcome, not only to avoid diagnostic surgeries and enable active surveillance, but also to guide the extension of thyroidectomy (total or partial), particularly in Bethesda categories III and IV nodules.

In this issue, Ferraz C. reviewed molecular tests developed to improve the diagnosis of indeterminate biopsies (2). A classification according to their predominant ability to “rule-in” and/or “rule-out” cancer was proposed. She pointed out that a desirable test to predict malignancy would have high positive predictive value, while prediction of benign nodules would require high negative predictive values.

It is not easy to critically analyze a molecular test to diagnose thyroid cancer. The performance of each molecular test should be based on its characteristics, given by sensitivity, specificity and predictive values. Sensitivity and specificity correspond to the rate of thyroid cancer and of benign nodules detected by the test. Positive predictive value (PPV) is the proportion of thyroid cancer among positive test results and negative predictive value (NPV) is the proportion of benign nodules (not cancer) among negative results, which are both dependent on the prevalence of cancer in the studied population. Therefore, if you consider the probability of thyroid cancer in Bethesda category III and IV around 25%, a good “rule-out” test would have sensitivity > 90% to obtain a NPV > 94%, and a good “rule-in” test would have specificity > 80% to result in a PPV > 60%.

The first molecular test commercially available in Brazil was DNA sequencing to detect *BRAF* mutation. We prospectively evaluated the presence of the p.V600E mutation of the *BRAF* gene, and also searched for *N-RAS, H-RAS, K-RAS* mutations, in FNAB of Bethesda categories III and IV (3). *BRAF* mutation was detected in 65% of carcinomas included in our analysis. This simple test had specificity of 100% and PPV of 100% in both Bethesda categories III and IV. However, sensitivities were low, 35% and 57%, resulting in NPVs of 81% and 86%, respectively. When you order *BRAF* mutation test, a “positive” result assures 100% chance of malignancy but, if “negative”, the nodule is still considered indeterminate and a diagnostic surgery is necessary.

Performance of such “rule in” test was improved by additional evaluation of *PAX8/PPARg, RET/PTC1, RET/PTC 3* rearrangements, “7-gene panel” (4), currently available as ThyGenX^®^. Despite improvement, particularly in Bethesda category III, independent clinical validation studies did not replicate the performance, and a desirable NPV to avoid surgery was not reached, as false negative results would occur in more than 5% of the cases.

As reviewed by Ferraz C., new technologies, especially the next generation sequencing (NGS), provided a significant step-forward to clinical acceptance of molecular tests in the preoperative evaluation of thyroid nodules (2). The Afirma^®^ gene expression classifier (GEC) differentiates benign and malignant nodules based on patterns of mRNA expression (5). It was proved to be a cost-effective “rule-out” test to avoid surgery due to high sensitivity and NPV around 95%. As benign results were obtained in 41% of evaluated nodules in a clinical validation study, it has been suggested to be also cost-saving, since almost one out of two molecular tests would avoid one diagnostic surgery. Afirma^®^ GEC has been extensively evaluated by different and independent centers. Similarly to seven-gene panel, post-validation trials usually did not submit all patients to surgery, as a matter of fact, most of the “benign” GEC did not undergo surgical intervention, which could mislead an excellent performance. The cost-saving capacity was not confirmed, since depending on patients’ selection, more tests were necessary to avoid one surgery. Besides, it became evident that the performance of the test relied on the prevalence of malignancy of the studied population. If the rate of malignancy was lower than 25%, the cost-effectiveness of Afirma^®^ GEC decreased, as fewer “suspicious” results corresponded to thyroid carcinomas. A novel Afirma^®^ gene sequencing classifier (GSC) has been recently developed to improve evaluation of RNA expression and GSC increased specificity, particularly to recognize more Hürthle lesions as “benign”, preserving its high sensitivity (6).

Some molecular tests were reported as being not only able to correctly identify most of thyroid carcinomas, but also most of the benign lesions called “rule-in and rule-out” tests. ThyroSeq^®^ v2 with expanded panel of mutations, rearrangements and gene expressions, particularly in Bethesda category IV, is apparently efficient in indicating surgery if “positive”, and to consider follow-up without diagnostic surgery if “negative” (7). Post-validation studies demonstrated the usefulness of ThyroSeq^®^ v2 molecular test to avoid surgeries, as most of “negative” results were not submitted to surgical procedures, confirming it as a good “rule-out” test. However, as regards Bethesda category III lesions, Thyroseq^®^ v2 presented poorer performance and did not prove to be such a good “rule-in and rule-out” test. More recently, Nikiforova and cols. developed a new version of ThyroSeq^®^ v3, which provides a genomic classifier (GC) score calculated according to the strength of association of detected genetic alterations with malignancy (8). Since the presence of a mutated gene is not synonymous of malignancy, Thyroseq^®^ v3 presents different reports for “negative” and “positive” results. There are two classes of “negative” results: “negative”, as expected to be a benign lesion and “currently negative”, when a mutation is found in a low-risk gene that by itself is not sufficient to full cancer development (i.e., mutation in *PTEN, EIF1AX*) or it is found in a subpopulation of cells. Although at the time of sampling most of these nodules are benign, some of them may undergo clonal expansion and acquire additional mutations, so active surveillance is suggested, considering to repeat FNA and, possibly, molecular testing after one year of follow-up. When test result is “positive”, the prognosis is promptly suggested: “low-risk”, when *RAS*-positive is found, and ‘high risk’, when *TERT* and *BRAF*-positive carcinoma are present. Therefore, multicenter clinical trials are necessary to validate its performance.

Finally, microRNA (miRNA) gene expression classifiers have also been developed to improve diagnostic performance of Bethesda categories III and IV, ThyraMIR^®^ combined with ThygenX^®^ (9), Rosetta GX Reveal^®^ (10) and the Brazilian mir-THYpe^®^ (11). Their main limitation is lack of multicenter experience. MicroRNAs panels should be more extensively studied in order to confirm their performance as desirable both “rule-in and rule-out*’*” tests.

A remark should be made about molecular tests in the recently proposed noninvasive follicular thyroid neoplasm with papillary-like nuclear features (NIFTP), which corresponds to the noninvasive encapsulated follicular variant of papillary thyroid carcinoma. Molecular tests available nowadays proved to be unable to classify NIFTPs as benign lesions. In retrospective evaluations, 81% out of 32 NIFTPs analyzed by Afirma^®^ GEC had “suspicious” results (12). Likewise, 3 out of 5 NIFTPs submitted to ThyroSeq^®^ v.2 had “positive” results (13). Meanwhile, treatment of NIFTP is still surgical removal due to the potential risk of progression to invasive carcinoma. Therefore, “suspicious” or “positive” results in “rule-in and/or rule-out”’ test will not change NIFTP management.

## WHICH TEST SHOULD BE CHOSEN?

Only a few institutions reported their experience and compared the performance of molecular tests in similar conditions. In one of them, two-thirds of Bethesda categories III and IV lesions were managed nonoperatively based on nonsuspicious results of Thyroseq^®^ v2 or Afirma^®^ GEC. Considering the rate of malignancy of 14%, Livhits and cols. (14) demonstrated that ThyroSeq^®^ v.2 had a better performance to identify malignancy compared to GEC (PPV 57% vs. 39%). Similarly, Jug and cols. demonstrated that “negative” results in molecular tests helped to reduce surgery indication in ~50% of patients (15). Considering the rate of malignancy of only 12%, ThyroSeq^®^ v.2 had PPV of 40% and 50% in Bethesda categories III and IV and GEC had PPV of 29% in Bethesda category III and 27% in Bethesda category IV. Therefore, the performance of the molecular tests must be carefully interpreted, considering that different populations, diverse prevalence of malignancy, and the fact that not all patients were submitted to confirmatory surgery altogether, interfere in results when compared to clinical validation studies. While microRNA panels have limited multicenter experience, we could suggest that both Afirma^®^ GEC/GSC and ThyroSeq^®^ v2/v3 might be used to improve preoperative diagnosis of Bethesda categories III and IV lesions. High cost and no health insurance coverage limit the widespread application of molecular tests in Brazil and other countries.

It is always important to consider risk factors, patient´s clinical conditions and desire, and, certainly, US characteristics before choosing a molecular test. We usually wish to identify benign lesions in order to defer diagnostic surgeries. High-risk nodules at US may not benefit from “rule-out” molecular test to avoid surgery. Actually, in high-risk nodules, a “positive” result in a “rule-in” test, reinforcing malignancy, is more useful, as a partial diagnostic surgery may turn into total thyroidectomy to treat cancer. On the other hand, if we evaluate an indeterminate or low-risk nodule at US, “rule-out*”* tests seem more relevant because of their low rate of false-benign results.

Implementation of molecular test into routine clinical practice should be made with cautious, as long-term outcome data on companion use of molecular test to guide therapeutic decision-making is currently lacking. To conclude, as *The American Thyroid Association* strongly recommends: “if molecular testing is being considered, patients should be counseled regarding the potential beneﬁts and limitations of testing and about the possible uncertainties in the therapeutic and long-term clinical implications of results” (1).
